# Restoration of Noncarious Cervical Lesions: When, Why, and How

**DOI:** 10.1155/2012/687058

**Published:** 2011-12-18

**Authors:** Cesar dos Reis Perez, Mariana Rodrigues Gonzalez, Natália Aráujo Silva Prado, Marianna Sorozini Ferreira de Miranda, Mariana de Andrade Macêdo, Bárbara Monteiro Pessôa Fernandes

**Affiliations:** School of Dentistry, State University of Rio de Janeiro (UERJ), Avenida 28 de Setembro, 157, Vila Isabel, 20551-030 Rio de Janeiro, RJ, Brazil

## Abstract

At this time, restoration of noncarious cervical lesions (NCCLs) is a common occurrence in clinics nowadays. Some reasons for this are the growth of the elderly population, a smaller rate of tooth loss, and possibly the increase of some etiologic factors. These factors include inadequate brushing techniques in gingival recession cases, corrosive food and drink consumption, and occlusal stress concentrating factors (occlusal interferences, premature contacts, habits of bruxism, and clenching). Unfortunately, Class V restorations also represent one of the less durable types of restorations and have a high index of loss of retention, marginal excess, and secondary caries. Some causes for these problems include difficulties in isolation, insertion, contouring, and finishing and polishing procedures. This work aims to help dentists in choosing the best treatment strategy, which necessarily involves steps of problem identification, diagnosis, etiological factor removal or treatment, and, if necessary, restoration. Finally, appropriate restorative techniques are suggested for each situation.

## 1. Introduction

Noncarious cervical lesions (NCCLs) are becoming an increasingly important factor when considering the long-term health of the dentition. In fact, the occurrence of this condition is steadily increasing [1–4]. According to the present literature available, it is not possible to determine a unique etiological factor, but there is a concern that it is a multifactorial condition [5–8]. These lesions can affect tooth sensitivity, plaque retention, caries incidence, structural integrity, and pulp vitality, and they present unique challenges for successful restoration [5–9]. These challenges involve each step of the restoration process, including isolation, adhesion, insertion technique, and finishing and polishing [10]. A successful diagnosis and treatment plan requires keen observation, a thorough patient history, and careful evaluation. This work aims to provide some knowledge of the NCCLs' characteristics and etiologic covariables as well as improve assessment of prognosis by aiding in proper case selection for treatment and in the selection of appropriate treatment protocols.

## 2. Identification of the Problem and Etiology

The first step for a successful treatment is the early identification of the problem. This could be reached with a complete patient anamnesis accompanied by a careful clinical examination. Some studies suggest that treatment provided for NCCLs may not be based on the correct diagnosis [3, 4]. It is important to diagnose the tooth wear process in children and adults as early as possible. Dental professionals have to rely on clinical appearance to diagnose erosion. Diagnosing early forms of erosion is difficult, as erosion is accompanied by few signs and fewer, if any, symptoms. Therefore, clinical appearance is the most important feature for dental professionals when diagnosing this condition. This is of particular importance in the early stage of dental erosion. The teeth should be dried thoroughly and well illuminated to note minor surface changes [5].

Commonly, when the NCCL is painless and does not affect esthetics, there is no complaint by the patient. Sometimes, it is not completely painless, but the dentin is partially (or completely) covered by dental plaque, tartar, or gum. A simple removal (or displacement) of this coverage followed by the application of some stimulus (like a delicate air blast) can initiate a pain process. When pain is present, the location of the lesion becomes easier to detect. Pain is one of the factors that will directly influence the decision for restorative therapy as well as the technique employed. 

As soon as the dental caries is eliminated as primary cause, the possible factors involved have to be identified. These noncarious processes may include abrasion, corrosion, and (possibly) abfraction, acting alone or in combination. 

There are factors associated directly with the genesis of NCCL, such as occlusion, saliva, age, sex, diet, and parafunctional habits [11, 12]. 

Abrasion is the result of friction between a tooth and an exogenous agent [13]. If teeth are worn on their occlusal surfaces, incisal surfaces, or both by friction from the food bolus, this wear is termed “masticatory abrasion”. Masticatory abrasion can also occur on the facial and lingual aspects of teeth, as coarse food is forced against these surfaces by the tongue, lips, and cheeks during mastication. We should not underestimate the relevance of some current diet habits, which are considered “healthy” but potentially destructive to the teeth (granolas, nuts, all bran cereal, and acid juices). Abrasion can also occur as a result of overzealous tooth brushing, improper use of dental floss and toothpicks, or detrimental oral habits. The NCCL, with prevalent influence of abrasion, often presents hallmarks. Frequently, they appear as painless cavities with polished surfaces, but pain is not an uncommon occurrence. Typically, when improper tooth brushing is one of the causes of the NCCLs, the enamel resists differently than the dentin which erodes following the path made by the toothbrush [3–9].

In dentistry, the term erosion is used to define the loss of dental hard tissues by chemical action not involving bacteria. Erosion, as defined by the American Society for Testing and Materials Committee on Standards [14], is “the progressive loss of a material from a solid surface due to mechanical interaction between that surface and a fluid, a multi component fluid, impinging liquid or solid particles.” Therefore, this terminology should be avoided in dentistry.

Corrosion is the more appropriate term and represents tooth surface loss caused by chemical or electrochemical action. There are both endogenous and exogenous sources of corrosion. In cases of endogenous sources of corrosion, such as bulimia or gastro esophageal reflux disease (GERD), the enamel appears thin and translucent, enamel is lost on the posterior occlusal and anterior palatal surfaces, and depressions occur at the cervical areas of upper anterior teeth. “Cupped,” or invaginated, areas develop where dentin has been exposed on the occlusal surfaces of posterior teeth because of wear. In the exogenous sources of corrosion, the aspect is similar, but the tissue loss location modifies following the areas related to the passage of the corrosive element [7]. It has been reported that any food substance with a critical pH value of less than 5.5 can become a corrodent and demineralize teeth. This may occur as a result of consuming highly acidic foods and beverages such as citrus fruits, carbonated soft drinks, and sucking on sour candies. Acidic mouthwashes also may be implicated. Acidulated carbonated soft drinks have become a major component of many diets, particularly among adolescents and young children. It is evident that this condition does not exclusively affect the cervical areas, but, in association with other factors, it will act synergistically [15]. 

Abfraction is thought to take place when excessive cyclic, nonaxial tooth loading leads to cusp flexure and stress concentration in the vulnerable cervical region of teeth. Such stress is then believed to directly or indirectly contribute to the loss of cervical tooth substance [5, 7, 8, 16–23]. 

Although there is theoretical evidence in support of abfraction, predominantly from finite element analysis studies, caution is advised when interpreting results of these studies due to their limitations [9, 24–26].

Frequently, more than two mechanisms may be involved in the etiology of tooth surface lesions, featuring a multifactorial phenomenon. For example, a corrosive cervical lesion could be exacerbated by tooth brushing abrasion. When to these two mechanisms are added the effect of stress (abfraction) resulting from bruxism or occlusal interference, these lesions then become corrosive-abrasive abfractive in nature. These various mechanisms can occur either synergistically, sequentially, or alternately. The interplay of chemical, biological, and behavioral factors is crucial and helps to explain why some individuals exhibit more erosion than others [5, 7]. Therefore, awareness of a multifactorial etiology in noncarious cervical lesions may help the clinician to formulate an appropriate treatment plan for the patient.

## 3. Removing (or Treating) the Causes

Abrasion is the most cited etiological factor for development of NCCL. In clinical surveys, 94% of respondent dentists classified the lesion as abrasion, and 66% rated tooth brushing as the most likely cause. The treatment methods used varied, with no clear preference [9]. Cervical toothbrush abrasions are generally thought to be a consequence of toothbrush factors such as frequent or forceful tooth brushing, faulty or vigorous techniques, filament stiffness or design, dominant hand dexterity, or abrasive dentifrices. However, investigations cannot conclusively establish one factor as the primary etiology because of conflicting results. Therefore, an array of aspects related to toothbrush factors may operate in conjunction with dental erosion and occlusal loading [15]. Nevertheless, this etiology can be controlled. The clarification of patients, their orientation about brushing techniques, and the change of some of the above factors can bring tangible results and must be performed.

Another etiology that can be effectively modified is the chemical corrosion (also called “dental erosion”) and should be correctly diagnosed. The success of the treatment depends upon the patient's collaboration. When derived from eating disorders (bulimia) or and GERD, the treatment may require the participation of a physician. The extrinsic etiology is more easily treatable; removing or altering the harmful habit, as in the abrasion etiology, provides consistent results.

When the abfraction etiology is diagnosed, no consensus on treatment strategies exists. It is important that oral health professionals understand that abfraction is still a theoretical concept, as it is not proved. As a result of the reported associations between occlusal interferences and abfraction lesions, and between loading direction (influenced by cusp inclines) and unfavorable tensile stresses, occlusal adjustment has been advocated to prevent their initiation and progression and to minimize failure of cervical restorations. Occlusal adjustments may involve altering cusp inclines, reducing heavy contacts, and removing premature contacts. The effectiveness of such treatment is not supported by evidence. In fact, inappropriate occlusal adjustments may increase the risk of certain conditions such as caries, occlusal tooth wear, and dentine hypersensitivity [24]. The science of occlusion is complex, and the treatment requires understanding, care, and experience. Although it is desirable to reduce lateral forces on teeth with stress-induced cervical lesions, extensive restorative procedures, such as the reestablishment of anterior guidance or orthodontic movement, require cost-and-benefit justification.

Occlusal adjustment should be undertaken only in cases where the interferences are well established and diagnosed. The professional must be enabled to do the adjustments and be aware that this procedure must be performed only when strictly indicated. The adjustment must be carried out in order to remove only the interferences, preserving the original points of centric occlusion. Another possibility exists: the creation of a protective canine guidance with composite resin. It is a conservative procedure, since it involves only the application of a composite resin, but it is important to carefully observe the possibility of excessive stress concentrated on this tooth.

In fact, it is recommended that destructive, irreversible treatments aimed at treating so-called abfraction lesions, such as occlusal adjustment, must be avoided or implemented only in exceptional cases.

Occlusal splints, aimed at reducing the amount of nocturnal bruxism and nonaxial tooth forces, have been recommended to prevent the initiation and progression of abfraction lesions. However, it should be noted that the use of occlusal splints to reduce bruxism is still a controversial topic. Some studies support their efficacy [18]. Occlusal splints have the potential to reduce nonaxial tooth loading when constructed appropriately. Although they provide a conservative treatment option for managing suspected abfraction lesions, according to some authors, there is no evidence base to support their use [9, 24]. In the presence of evidence of the relevance of the abfraction mechanism in the development of lesions, the occlusal splint should be considered as a good treatment strategy due to its conservative nature.

### 3.1. Accompaniment

It should be noted that when restoring NCCLs, clinicians are not treating the etiology but are merely replacing what has been lost. Some dentists recommend watching and waiting. Others recommend early intervention [6, 16, 24, 26, 27]. There are no generally accepted, specific guidelines in the literature stating that all lesions should be restored. Logic and good clinical judgment would suggest that they should be restored when clinical consequences (e.g., dentine hypersensitivity) have developed or are likely to develop in the near future. Aesthetic demands of the patient may also influence the decision to restore these lesions. One must conduct a risk-benefit analysis when considering restoring these lesions. Cervical restorations may contribute to increased plaque accumulation potentially leading to caries and periodontal disease [11, 24, 25]. 

There are different reasons for the need for restorative treatment: the structural integrity of the tooth is threatened, the exposed dentin is hypersensitive, the defect is esthetically unacceptable to the patient, or pulp exposure is likely to occur [5]. 

When the dentist is against nonsensitive shallow cavities that do not provide additional plaque retention, accompaniment should be performed. The possible causes of the NCCLs should be identified and eliminated (or treated). 

Photographic records should be taken annually as well as full-arch impressions. The models should be kept safe for future comparisons. If the abfraction etiology is considered, the occlusion should be marked with red and blue articulating paper to check whether there has been any change, and photographic records from an occlusal view should be taken. If a progression of the NCCLs is diagnosed, changes in the therapy should be considered, providing restorative treatment if necessary [6, 19].

### 3.2. Restorative Treatment

Once the restorative treatment is indicated, the dentist has to know the different causes and aspects of each situation and choose the best strategy to employ. Unfortunately, although NCCL restorations are a very common occurrence in clinics, they also represent one of the less durable types of restorations and have a high index of loss of retention, marginal excess, and secondary caries [10]. Despite these restorations being a continuing problem in restorative dentistry, the causes of the diminished longevity are still poorly understood. Failure of cervical adhesive restorations is often attributed to inadequate moisture control, adhesion to different opposite substrates (enamel and dentin), differences in dentin composition, and also cusp movement during occlusion. In order to help adopt the best restorative strategy, each step of the restorative procedure will be considered.

### 3.3. Isolation

Problems with restoring NCCLs include difficulty in obtaining moisture control and gaining access to subgingival margins [10, 28–30]. Rubber dam clamps, gingival retraction cord, and periodontal surgery are methods that can be used to retract and control the gingival tissues, and thus facilitate access and also control moisture. The exudation of gingival fluid is possibly one of the challenges to adhesion in cervical region, which is already impaired by other factors (such as the absence of enamel in the gingival wall of the cavity and the characteristics of the dentin in NCCLs). Rubber dam isolation should be used whenever possible. Intrinsic anatomical and morphological characteristics of the cervical region create limitations in the placement of the rubber dam and clamp. Proper isolation, is very difficult, sometimes impossible, when lesions extend proximally or under the gingiva. Sometimes part of the structure cannot be isolated and the dam promotes restorative material accumulation. Access is also limited, causing problems related to insertion of the restorative. When adequate rubber dam isolation is not possible another isolation method has to be employed. The insertion of nonimpregnated retraction cords can help in moisture control. Another option is a proposed association of Mylar matrix with wood wedges and a photocured gingival barrier [10]. In any case, a proper isolation is the first step for the success in restoring NCCLs but, despite being the basis for the other subsequent steps, is probably the most underestimated one.

### 3.4. Material Selection

Even with advanced destruction, minimally invasive restorative intervention, such as sealing or covering with composite material, should be the therapy of choice. It is evident from the recent literature that there is no place for metallic materials such as amalgam and gold in the modern day restoration of NCCLs. Glass ionomer cements (GICs), resin-modified GICs (RMGICs), a GIC/RMGIC liner base laminated with a resin composite, and resin composite in combination with a dentine bonding agent are all restorative options [24, 31–35].

Some authors recommend that RMGIC should be the first preference for restoration of NCCLs or, in aesthetically demanding cases, a GIC/RMGIC liner base with resin composite [32, 33]. Indeed GIC presents several characteristics that make them a good choice: biocompatibility, adhesion to calcified substrates (especially in cases of dentin sclerosis where traditional adhesion may underperform), and elastic modulus similar to the dentin. However, some other characteristics make its use infrequent: technical difficulties related to the material's stickiness, poor esthetics, solubility particularly in acidic oral environments, and retention failure occurrences. Some authors claim that under the action of parafunctional loadings, fracture-induced failure of cervical GIC restorations occurs at the cervical margin. It is further shown that prior to fracture, the restorative material undergoes strain softening, which in turn introduces damage and weakens the materials involved. The softening of the material occurs in the cervical region of the restoration area which has been linked to the location of most of the clinical observed failures [30]. This can be related to the brittleness of the material (cement). The author does not indicate GIC or RMGIC frequently, but it is a good indication in deep NCCLs, where a laminate technique (sandwich technique with composite resins) can be used. 

The best materials for restoration of NCCLs are the composite resins. Within this group of materials, some authors recommend that NCCLs suspected of being caused primarily by abfraction should be restored with a microfilled resin composite or a flowable resin that has a low modulus of elasticity, as it will thus flex with the tooth and not compromise retention [34, 36–38]. However, no definitive conclusion can be found in the literature addressing the difference between failures rates of resin composites of different stiffness used to restore NCCLs. Nevertheless, in must situation, the authors recommend low modulus composites or associations of composites with different modulus [10].

### 3.5. Cavity Cleaning

After the isolation another important, and commonly neglected, step should be performed: the prophylaxis of the cavity. Due to their nature, NCCLs are lined with a contaminated layer that resists adhesion. The gingival proximity (sometimes partially or totally covering the cavity) makes this procedure a more complex step. In some cases, rotary prophylactic brushes cannot be used in order to avoid mechanical aggression and bleeding [10]. 

In nonsensitive cavities, the authors recommend rubbing the cavity and its periphery with a cotton pellet soaked with an anionic detergent, followed by rinsing with water, drying, and conventional total acid etching (37% phosphoric acid—10 seconds on dentins and 20 seconds on enamel) with the aim of removing the sticky layer. Even when the roughening procedure is performed, the same sequence is recommended.

In the presence of sensitivity, rubbing with detergent is still indicated but the phosphoric acid should be applied only on enamel. Dentin will be conditioned by the self-etching primer/adhesive. When a conventional GIC is chosen, the previous conditioning with polyacrylic acid is indicated in order to provide a good surface wetting. If an RMGIC is chosen, pretreatment of dentin with self-etch adhesive systems, before filling, seems to be a good alternative to the conventional dentin conditioner provided by the manufacturer [35].

### 3.6. Adhesion

Some intrinsic characteristics of the NCCL create unique challenges to dental adhesion. Some recent studies demonstrate important histological differences between prepared dentin and the affected dentin from NCCLs. 

One work based on Raman analysis showed that the distinct compositional and structural alterations in mineral and matrix components of NCCLs affected dentin. A heterogeneous hypermineralized layer, with characteristic features such as high phosphate/low carbonate content, high degree of crystallinity, and partially denatured collagen, was revealed in the affected dentin substrate of NCCLs [39, 40].

In another study focusing on adhesion to sclerotic dentin, the authors observed that most dentinal tubules were obliterated by rod-like sclerotic casts and could not be dissolved by acid etching. Both the hybrid zone and the resin tags were observed in sclerotic dentin after restoration. Although resin tags were fewer, and in lack of communications, the length of resin tags and the thickness of the hybrid zone were almost similar to those of the sound dentin. They concluded that bonding to sclerotic dentin is different from bonding to sound dentin and may be compromised by fewer resin tags and communications [41]. 

Transmission electron microscopy revealed that in addition to occlusion of the tubules by mineral crystals, many parts of wedge-shaped cervical lesions contain a hyper mineralized surface that resists the etching action of both self-etching primers and phosphoric acid. This surface prevents hybridization of the underlying sclerotic dentine. In addition, bacteria are often detected on top of the hypermineralized layer. Acidic conditioners and resins penetrate variable distances into these multilayered structures. Examination of both sides of the failed bonds revealed a wide variation in fracture patterns that involved all of these structures. Microtensile bond strengths to the occlusal, gingival, and deepest portions of these wedge-shaped lesions were significantly lower than similar areas artificially prepared in normal teeth [42].

Further studies are required to understand the role that these alterations play in response to acid etching and bonding to these clinically relevant substrates. 

Further, some authors agree that restorations placed in teeth whose dentin/enamel had been prepared, or roughened, showed a statistically significant higher retention rate than those placed in teeth with unprepared dentin [10, 43]. Considering these studies and the author's clinical experience, a mild roughening of the superficial dentin with a diamond point is indicated when restoring polished nonsensitive NCCLs. This procedure does not create additional sensitivity and aims to get a more reliable adhesion in this specific situation. If the cavity is deep and provides sufficient thickness, a sandwich technique may be performed, taking advantage of the GIC's good adhesion to calcium. It is important to note that adhesives with direct interaction with calcium have been recently developed and present a promising option in these cases [43]. 

The adhesion strategy for sensitive NCCLs has to be different. Using common sense, it is logical to conclude, based on hydrodynamic theory, that the dentin tubules are not obliterated; on the contrary, they are probably opened. Thus, the etching should be gentle in order to provide a good substrate to adhesion without enhancing sensitivity.

Based on this, and considering the available adhesives, the self-conditioning (SE) adhesives should be the first choice. Although several articles doubt their efficiency in aspects such as bond strength and marginal discoloration [44], others demonstrate acceptable clinical performance [45–49]. A previous acid etching of the surrounding enamel is indicated because, as known, the microretentions created by the SE adhesives are not enough to give adhesive strength similar to that achieved by conventional acid etching. Within this group, the self-etching primers (two steps) present better results than the self-etching adhesives (one step) [50–52]. One must always remember that an active application of these adhesives should be employed, rubbing the surface with a soaked microbrush for 15-seconds, waiting other 15 second period to allow volatilization of solvents. This is important because the cervical wall of the cavity tends to retain excess of adhesive which leads to future discoloration and gap formation.

### 3.7. Insertion Techniques

Despite the apparent easy access and insertion, NCCL presents some particularities that should be emphasized. This may justify the high documented failure rate [30, 33, 53–55] and the number of published articles about this theme [10, 34, 36, 56–67].

The first point that creates difficulties is that the cavity limits are not well defined, especially the proximal limits location. Thus, restorations with excess material are a common occurrence. Every effort should be made to delimit the future restoration, because the excess removal and the finishing and polishing present other difficulties. A good gingival displacement and the use of enhancing optical devices are indicated. 

Another challenge is eliminating or reducing the gap formation on the gingival wall. The simple fact of working with cavities on opposite walls from dissimilar tissues like dentin and enamel already creates intrinsic problems. Managing their completely different adhesive behavior is one aspect that should not be overlooked.

Several restorative techniques have been proposed to minimize shrinkage due to polymerization and also to achieve better marginal adaptation in Class V cavities. Because bond strength to enamel is usually greater than to dentin, it was suggested that cavities could be restored in multiple layers, starting with incremental placement in the occlusal wall of the preparation. This would minimize leakage into the dentin margin. It has also been suggested that the contraction gap at the gingival margin caused by polymerization shrinkage could be prevented by the incremental placement of a composite material starting in the dentin portion of the preparation. Regarding the possibility of bulk placement, it has been stated that this often results in open dentin margins, thus increasing microleakage [10].

 Since enamel adhesion is stronger, more stable, and more predictable, the insertion of material should begin from the gingival wall, without surrounding enamel. Avoiding concomitant insertion on opposite walls and leaving a free surface, the adhesion to the cervical wall can be achieved without antagonistic forces. Whenever possible, the cavity should be restored with three, or at least two, increments. The last one will be placed on the enamel margin. Employing a careful technique is possible to achieve a restoration with minimum or no finishing and polishing procedures needed.

Considering esthetics, the color of the cervical area is easy to obtain, usually with a higher saturation and smaller translucency compared to the color of the other two thirds of the tooth.

### 3.8. Finishing and Polishing

Any excess or roughness should be avoided in NCCLs' restorations. Plaque retention, gingival inflammation, and occurrence of caries lesions represent not only a failure of the restoration but also a creation of new problems to the patient. Poorly performed finishing and polishing procedures can lead to damage to the soft and hard tissues. Techniques with minimum need of finishing and polishing are ideal, but properly contoured restorations are seldom achieved without the need to remove excess material [10, 68–72]. When they are needed, a good option is the use of delicate diamond finishing points followed by application of a surface sealant or a liquid polisher [10, 72, 73].

### 3.9. Clinical Control

As emphasized before, treatment of NCCLs is not easy, and sometimes, new procedures or different approaches are needed. Semiannual appointments should be performed in order to observe the evolution of the lesions, the conditions of the restorations, and other concerns of the patient. Also, the maintenance of the surface polish can be performed with a new surface sealant application.

## 4. Conclusions

Treating NCCLs necessarily involves these steps: problem identification, diagnosis, etiological factor removal, or treatment, and, if necessary, restoration. Due to the multifactorial character, it is not a simple procedure. A successful diagnosis and treatment plan requires a thorough patient history and careful observations and evaluations. Different approaches should be made to each specific situation.
